# Revisiting the use of adenosine deaminase in cerebrospinal fluid analysis for infectious meningoencephalitis

**DOI:** 10.1055/s-0046-1829158

**Published:** 2026-10-27

**Authors:** Luis Arthur Brasil Gadelha Farias, Osvaldo Mariano Viana Neto, Ednaldo Pereira Lima Sobrinho, Giuliana de Fátima Lima Morais, Nathalia Camila Maciel Jenkins, Deborah Nayara Santos de Farias, Bruno Cavalcante Fales de Brito Alves, Aldenise de Olinda Castro, Alex de Assis Santos dos Santos, Paulo Germano de Carvalho, Tania Mara Silva Coelho, Silvia Figueiredo Costa, Maura Salaroli de Oliveira, Lauro Vieira Perdigão Neto

**Affiliations:** 1 Universidade de São Paulo, Faculdade de Medicina, Hospital das Clínicas, Departamento de Doenças Infecciosas e Parasitárias, Laboratório de Investigação Médica em Protozoologia, Bacteriologia e Resistência Antimicrobiana (LIM 49), São Paulo SPBrazil.; 2 Hospital São José de Doenças Infecciosas, Fortaleza CEBrazil.; 3 Centro Universitário Christus, Fortaleza CEBrazil.; 4 Universidade Federal do Ceará, Fortaleza CEBrazil.; 5 Instituto de Bioestatística de Ciência e Tecnologia, Belém PABrazil.; 6 Secretaria de Saúde do Estado do Ceará, Fortaleza CEBrazil.; 7 Universidade de São Paulo, Faculdade de Medicina, Hospital das Clínicas, Serviço de Controle de Infecção Hospitalar, São Paulo SPBrazil.; 8 Hospital Sírio-Libanês, Serviço de Controle de Infecção Hospitalar, São Paulo SPBrazil.

**Keywords:** Adenosine Deaminase, Biomarkers, Meningoencephalitis, Cerebrospinal Fluid

## Abstract

**Background:**

Adenosine deaminase (ADA) levels in the cerebrospinal fluid have neither been validated as a reliable diagnostic marker nor sufficiently studied in patients with tropical diseases and human immunodeficiency virus (HIV) infection.

**Objective:**

To investigate cerebrospinal fluid (CSF)ADA levels in different central nervous system (CNS) infections, define a cutoff value, and assess correlations between CSF-ADA and other laboratory parameters.

**Methods:**

In 206 patients whose CSF-ADA levels were measured, we analyzed data on baseline characteristics, diagnoses, CSF-ADA levels, and additional laboratory parameters. These CSF-ADA levels were compared according to microbiological diagnosis, and correlations with other CSF and blood parameters were analyzed.

**Results:**

Compared with the non-CNS infectious control group ( *n* = 40), 2 subgroups showed significantly higher CSF-ADA levels: *Mycobacterium tuberculosis* ( *n* = 45, *p* < 0.0001) and *Neisseria meningitidis* ( *n* = 11, *p* = 0.0400). The identified cutoff values were 5.5 and 7.3 IU/L for *M. tuberculosis* and CNS coinfections, respectively. For *M. tuberculosis* , sensitivity was 61.2% and specificity was 72.4%, whereas coinfections demonstrated 50% of sensitivity and 82.8% of specificity. The CSF-ADA levels correlated positively with CSF protein (Spearman rank correlation coefficient [rs] = 0.3009, *p* < 0.0003) and weakly correlated negatively with CSF glucose (rs = -0.1895, *p* < 0.0254). Patients with *M. tuberculosis* infection exhibited significantly higher CSF-ADA levels, as reflected by the mean, median, and standard deviation, than those with other infectious etiologies combined ( *n* = 121) and those with bacterial and viral infections analyzed separately ( *p* < 0.05). When stratified according to HIV status, a significant difference in CSF-ADA levels was observed only among patients with varicella-zoster virus infection ( *p* = 0.0428).

**Conclusion:**

The CSF-ADA levels were elevated in tuberculous and meningococcal meningitis. Higher cutoff values may occur in CNS coinfections. Therefore, CSF-ADA may serve as a supportive diagnostic tool in cases of suspected TBM, meningococcal meningitis, and CNS coinfections.

## INTRODUCTION

Laboratory biomarkers facilitate early and accurate diagnosis of central nervous system (CNS) infections. The level of adenosine deaminase (ADA) in the cerebrospinal fluid (CSF) may help differentiate infectious etiologies, particularly in cases with overlapping clinical features. The ADA is a purine-degrading enzyme with neuroprotective functions that reflects CNS cellular immune activation. 1 2 The quantification of ADA in body fluids is simple, rapid, cost-effective, and can be performed using different laboratory methods. 2

The ADA assay is a well-established indirect method for diagnosing tuberculosis (TB) in body fluids, including pleural and pericardial effusions, ascites, and CSF, and is frequently used to differentiate TB from other conditions. 2 3 However, the diagnostic performance of ADA varies widely across studies and body fluids. 4 Although ADA has been validated in fluids such as pleural effusions, its reliability in the CSF remains unclear. Meta-analyses have not established a definitive ADA threshold for the diagnosis of tuberculous meningitis (TBM), and the results remain inconsistent. 2 4 A meta-analysis suggested broad ranges of ADA levels with variable diagnostic accuracy; however, none of the proposed cutoffs distinguished TBM from pyogenic meningitis. 4

Both human immunodeficiency virus (HIV) infection and malignancies may reduce ADA levels and limit its reliability. 5 Although ADA remains a widely used and validated test for pleural tuberculosis, 3 its utility in CSF infections—including TBM, fungal, bacterial and viral meningitides—remains unclear. In this study, we aimed to describe ADA levels based on the etiology, define cutoff values, and compare ADA levels with other CSF markers.

## METHODS

### Study design

This retrospective study involved an evaluation of data from the medical records of patients who were initially suspected of having a CNS infection and were admitted to a public tertiary infectious disease hospital in Northeast Brazil between January 2017 and December 2024. Clinical, epidemiological, and etiological diagnoses were analyzed.

Additional laboratory data (such as basic serum biochemistry and complete blood count) were included when available. The CSF parameters included ADA, white blood cell (WBC) count, and protein and glucose levels. For patients with more than one CSF-ADA level measurement, only the first result was considered. Furthermore, the CSF-ADA levels were considered only during hospitalization and before antibiotic initiation. Patients with confirmed CNS infections constituted the infection group; patients with noninflammatory headaches and normal CSF findings constituted the control group.

### ADA methods

Throughout the study period, CSF-ADA levels were assayed using a commercially-available kit (Ebram Produtos Laboratoriais).

### Definition and diagnostic methods for CNS infections

Meningitis was defined as the presence of clinical signs (such as fever, headache and neck stiffness) along with CSF abnormalities, as described by Pannu et al. 6 7 Encephalitis was defined as an altered mental status lasting ≥ 24 hours without an alternative cause and associated with at least 2 supportive findings: fever, seizures, focal neurological signs, CSF pleocytosis, neuroimaging, or electroencephalography (EEG) abnormalities. Meningoencephalitis was considered when features of both meningitis and encephalitis were present. 7 8

### Inclusion criteria and microbiological diagnosis

Patients with CNS infections who presented with clinical features of meningitis, encephalitis, or meningoencephalitis, along with CSF-ADA measurement at hospital admission, and microbiological confirmation by molecular methods, culture, CSF serology, or venereal disease research laboratory (VDRL) testing were included in the study. *Treponema pallidum* infection was defined as CSF-VDRL positivity and/or serum VDRL detection with abnormal CSF findings. 9 10

Etiological diagnosis was based on direct microscopic detection of organisms suggestive of CNS infection using India ink staining or bacterioscopy. In addition, CSF cultures for pyogenic bacteria (after suspected cases of contamination were excluded), as well as fungal and mycobacterial cultures, were performed. Moreover, the FilmArray meningitis/encephalitis (ME) panel (BioFire Diagnostics, LLC,) was used for molecular identification of a broad range of pathogens in CSF. For TBM, molecular diagnosis was performed using GeneXpert (Cepheid) and Xpert Ultra (Cepheid) assays. Cryptococcal infection was confirmed using a CrAg Lateral Flow Assay (Immuno-Mycologics) in CSF. 11

The control group included patients with primary noninflammatory headache disorders, such as migraine, as well as psychiatric and cerebrovascular disorders, with negative molecular and culture test results. All patients in the control group had normal CSF analysis results, including biochemical parameters and cell count, and their ADA levels were quantified.

### Data analysis

Clinical and laboratory data were entered into Microsoft Excel 2016. All statistical analyses were performed using IBM SPSS Statistics for Windows (IBM Corp.) software, version 15.0. Two analyses were performed to understand the ADA value in CNS infections:

- The initial cutoff point for CSF-ADA was established using a screening test, followed by the calculation of predictive values using the infection and control groups as references. The cutoff point was determined by applying an algorithm that compared the accuracy, sensitivity and specificity of all possible points; the point with the highest accuracy was set as the cutoff point. A receiver operating characteristic (ROC) curve was constructed to determine the best diagnostic performance of CSF-ADA levels. Group comparisons were performed using Kruskal-Wallis test with Dunn's post hoc testing; correlations were assessed using Spearman's correlation coefficient. A significance level of α = 0.05 was predetermined for rejection of the null hypothesis.
- The CSF-ADA levels in patients with TBM were compared with those who had other infectious etiologies. For each group, the mean, median, standard deviation, and interquartile range (IQR) of the CSF-ADA levels were calculated. The analyses were subsequently stratified according to HIV infection status.

## RESULTS

### Patient characteristics

Of the 1562 patients screened, 989 had confirmed CNS infections. Among these, CSF-ADA results were available for 170 patients. After excluding 4 patients without CSF abnormalities, 166 remained in the infection group (150 men [91.4%] and 16 women [9.6%]). Forty patients without CNS infection formed the control group. Among patients in the CNS infection group, the mean age was 41.4 years. Moreover, HIV infection was confirmed in 104 (62.6%) and 29 (72.5%) patients in the infection and control groups, respectively.

A total of 45 patients (27.1%) were diagnosed with TBM, with definitive microbiological confirmation. The infections diagnosed were attributable to fungi: *Cryptococcus* spp. ( *n* = 20) and *Histoplasma capsulatum* ( *n* = 2); bacteria: *Mycobacterium tuberculosis* ( *n* = 45), *Streptococcus pneumoniae* ( *n* = 23), *Treponema pallidum* ( *n* = 13), *Neisseria meningitidis* ( *n* = 11), *Streptococcus agalactiae* ( *n* = 1), *Streptococcus equi* ( *n* = 1), *Streptococcus suis* ( *n* = 1), *Streptococcus salivarius* ( *n* = 1), and *Leptospira interrogans* ( *n* = 1); viruses: varicella-zoster virus (VZV; *n* = 10), cytomegalovirus (CMV; *n* = 7), Chikungunya virus (CHIKV; *n* = 1), enterovirus ( *n* = 1), herpes simplex virus type 1 (HSV-1; *n* = 1), herpes simplex virus 2 (HSV-2; *n* = 1), human herpes virus 6 (HHV-6; *n* = 1), dengue virus (DENV; *n* = 1), and Zika virus (ZIKV; *n* = 1); and coinfections: CMV/VZV ( *n* = 4), *M. tuberculosis/T. pallidum* ( *n* = 3), VZV/ *T. pallidum* ( *n* = 2), CMV/ *Cryptococcus* spp./HHV-6 ( *n* = 2), CMV/ *Cryptococcus* spp. ( *n* = 1), *N. meningitidis* / *Cryptococcus* spp. ( *n* = 2), *M. tuberculosis* / *Cryptococcus* spp. ( *n* = 1), VZV/CHIKV ( *n* = 1), VZV/HHV-6 ( *n* = 1), VZV/ *H. capsulatum* ( *n* = 1), CMV/ *M. tuberculosis* ( *n* = 1), HSV-1/CHIKV ( *n* = 1), enterovirus/ *T. pallidum* ( *n* = 1), *M. tuberculosis* / *Candida parapsilosis* ( *n* = 1), and *M. tuberculosis* / *Candida tropicalis* / HHV-6 ( *n* = 1).

### CSF-ADA cutoff point

The CSF-ADA cutoff point (≥ 5.5 IU/L) was determined using a screening test in the infection ( *n* = 166) and control ( *n* = 40) groups. The test yielded the following performance metrics: sensitivity of 47.6%, specificity of 72.4%, and an overall accuracy of 51.2%.

The best diagnostic performance was observed in the mycobacterial subgroup ( *p* < 0.0001), with moderate sensitivity (61.2%), acceptable specificity (72.4%), and a cutoff value of 5.5 IU/L. In the coinfection subgroup ( *p* = 0.0269), moderate sensitivity (50%) and high specificity (82.8%) were observed at a cutoff value of 7.3 IU/L. Table 1 summarizes the CSF-ADA cutoff values stratified by etiological subgroup.

**Table 1:** CSF-ADA levels and cutoff values according to CNS infection subgroups

CSF-ADA (UI/L)								
Etiological group	n	Mean	SD	Median	Cutoff	Sensibility	Specificity	*p* -value
CNS infection	166	5.05	8.69	2.13	5.50	0.476	0.775	0.2475
Infection	144	4.95	7.11	8.12	5.50	0.473	0.724	0.3084
Coinfection	22	8.00	7.15	6.20	7.30	0.500	0.828	0.0269*
Virus	25	2.90	6.09	8.01	6.20	0.360	0.759	0.6333
Bacteria	52	3.60	6.49	8.85	5.50	0.423	0.724	0.4858
*Mycobacterium tuberculosis*	45	7.00	8.90	8.51	5.50	0.612	0.724	< 0.0001*
Fungi	22	4.75	5.77	4.52	5.20	0.500	0.690	0.5878
Control group	40	4.08	2.73	3.90				

Abbreviations: CNS, central nervous system; CSF-ADA, cerebrospinal fluid adenosine deaminase; SD, standard deviation.

Note: Mann-Whitney U test (Infectious etiology x control group).* *p* -value < 0.05.

### Comparison of CSF-ADA levels among infectious etiologies

Figure 1 illustrates CSF-ADA levels in patients with different infectious etiologies. The Kruskal-Wallis test, followed by Dunn's post hoc analysis, revealed a significant increase in CSF-ADA levels among patients with *M. tuberculosis* ( *p* < 0.0001) and *N. meningitidis* infections ( *p* = 0.043) compared with the control group.

**Figure 1:**
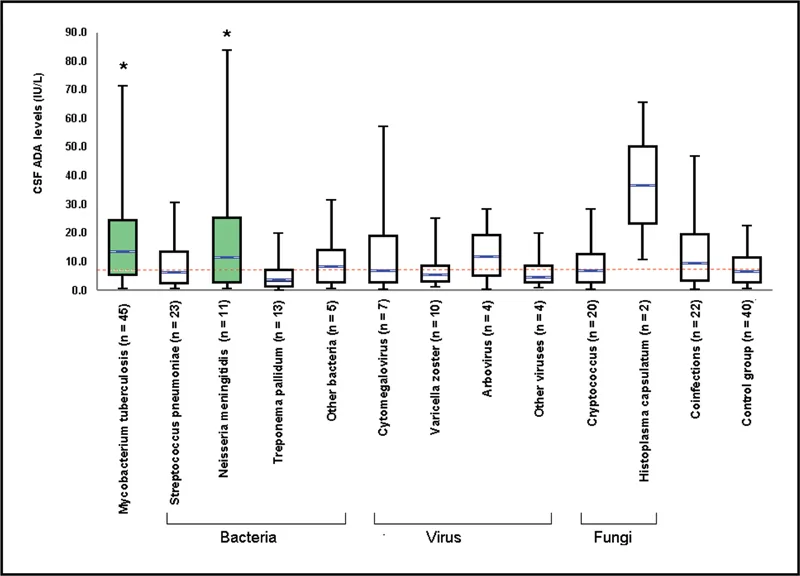
Notes: The boxplot markers represent the minimum, first quartile, median, third quartile, and maximum values. Green-shaded boxes indicate subgroups with a statistically significant increase in ADA levels compared with the control group, while white-filled boxes indicate subgroups without a significant increase. Asterisks (*) denote *p* -value < 0.05. The red dashed line represents the cutoff value (5.5 UI/L). Comparison of cerebrospinal fluid (CSF) adenosine deaminase (ADA) levels among different etiologies.

### Percentage increase in CSF-ADA levels among various etiologies

Figure 2 presents the proportion of patients with elevated CSF-ADA levels (≥ 5.5 IU/L) across different infectious etiologies. Notably, the two patients with *H. capsulatum* infection had increased CSF-ADA levels. The highest proportions of patients with elevated ADA levels were observed among those with arboviral infections (75%), *M. tuberculosis* (66.7%), *N. meningitidis* (63.9%), and coinfections (50%).

**Figure 2:**
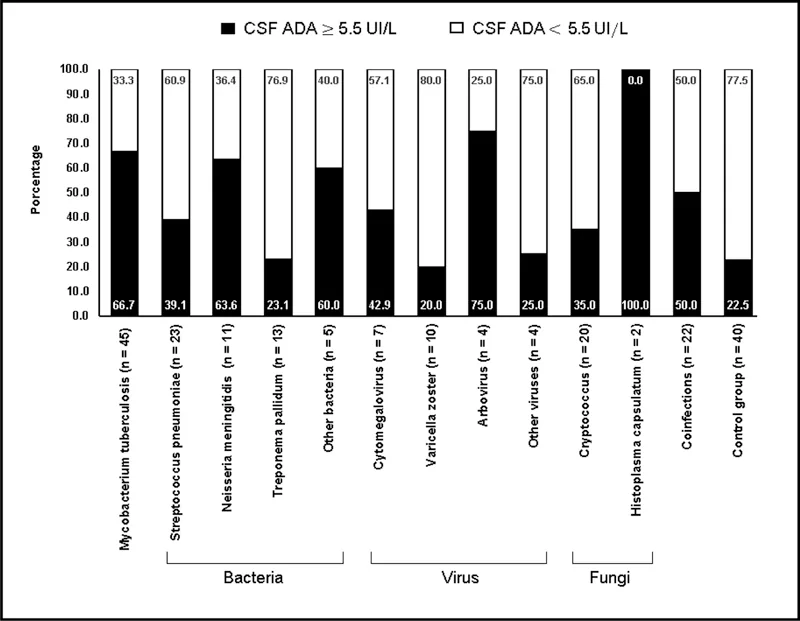
Percentage increase in CSF-ADA levels (≥ 5.5 IU/L) among various infectious etiologies.

### Median CSF-ADA levels across different infectious etiologies

*H. capsulatum* demonstrated the highest median ADA level (13.5 IU/L), followed by *N. meningitidis* (8.5 IU/L), *M. tuberculosis* (8.0 IU/L), and arboviruses (6.7 IU/L). In contrast, lower median ADA levels than those in the control group were observed in infections that were caused by other viruses (1.9 IU/L), *T. pallidum* (2.2 IU/L), VZV (2.2 IU/L), and *S. pneumoniae* (3.9 IU/L). Figure 3 illustrates the correlation between median CSF-ADA levels and the etiological diagnosis.

**Figure 3:**
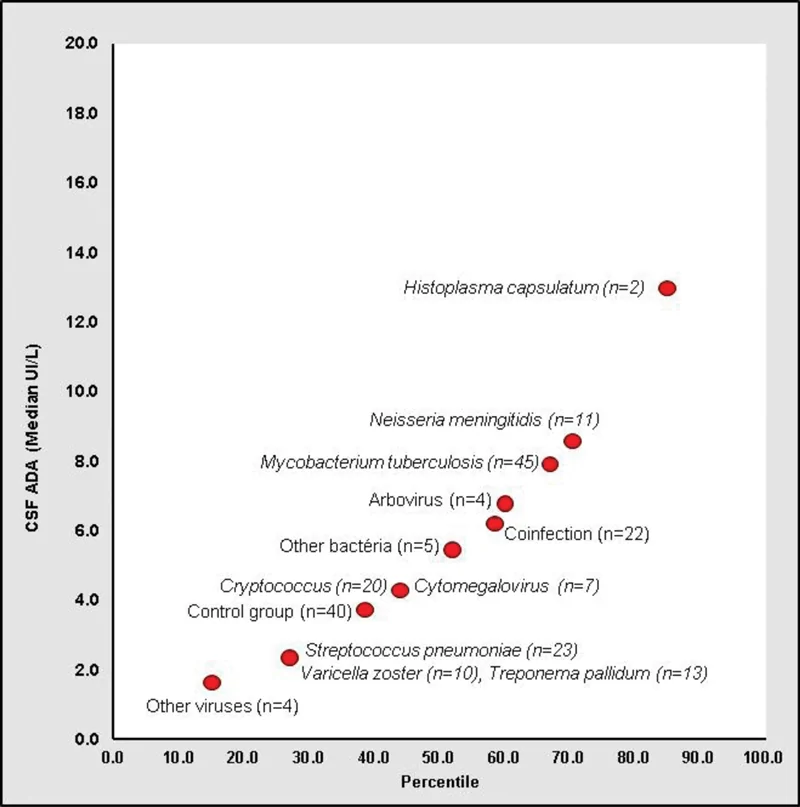
Median CSF-ADA levels (IU/L) according to infectious etiology ( *n* = 166).

Despite its higher median ADA level (8.5 U/L), the *p* -value of 0.0433 suggests a moderate association of *N. meningitidis* with ADA levels. *S. pneumoniae* presented lower ADA values (median: 3.9 U/L) with a nonsignificant *p* -value. Table 2 reveals the CSF-ADA diagnostic performance, predictive values, and ROC analysis across infectious etiologies. The ROC curve analysis across all etiological diagnoses demonstrated that *M. tuberculosis* infection had superior diagnostic performance compared with the other groups ( Figure 4 ).

**Table 2:** CSF-ADA diagnostic performance, predictive values, and ROC analysis across infectious etiologies

Etiological group	Etiology	Cutoff point	Sensitivity	Specificity	*p* -value	d	AUC	95%CI
**Mycobacteria**	*Mycobacterium tuberculosis*	5	0.711	0.725	< 0.0001*	0.4	0.718	0.62 to 0.81
**Bacteria**	*Streptococcus pneumoniae*	5.7	0.391	0.775	0.4959	0.65	0.583	0.54 to 0.61
	*Neisseria meningitidis*	5.7	0.636	0.775	0.0433*	0.43	0.705	0.67 to 0.73
	*Treponema pallidum*	3.1	0.615	0.65	0.2447	0.52	0.632	0.60 to 0.66
	Other bacteria	5.5	0.6	0.775	0.5753	0.46	0.6875	0.65 to 0.72
**Virus**	Cytomegalovirus	4	0.714	0.525	0.3572	0.55	0.6195	0.58 a 0.65
	varicella-zoster virus	1.6	0.9	0.25	0.4335	0.76	0.575	0.54 a 0.60
	Arbovirus	6.2	0.702	0.701	0.3525	0.42	0.7015	0.66 a 0.73
	Other viruses	3.8	0.703	0.702	0.5851	0.42	0.7025	0.66 a 0.73
**Fungi**	*Cryptococcus* sp.	5.2	0.45	0.75	0.4431	0.03	0.9825	0.97 a 0.99
	*Histoplasma capsulatum*	10.7	0.99	0.975	n/a	0.03	0.9825	0.97 a 0.99
**Coinfection**	**-**	7.3	0.5	0.85	0.0669	0.52	0.675	0.64 a 0.70

Abbreviations: AUC, area under the curve; CSF-ADA, cerebrospinal fluid adenosine deaminase; d, distance; ROC, receiver operating characteristic.

Notes: Mann-Whitney U test. The “d” represents the distance from each point on the ROC curve to the gold standard (the ideal point). Thus, the point with the lowest “d” value corresponds to the cutoff with the best overall diagnostic performance. * *p* -value < 0.05.

**Figure 4:**
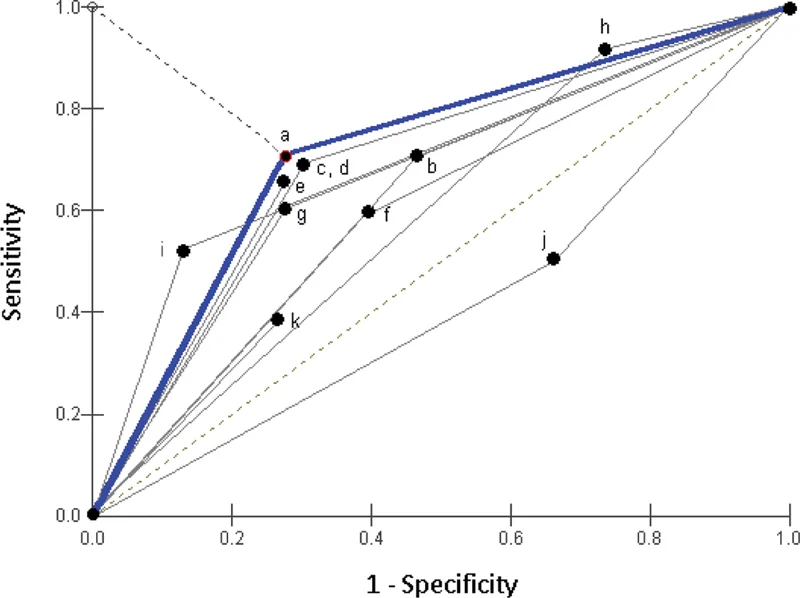
Receiver operating characteristic (ROC) curve of CSF-ADA levels for differentiating infectious etiologies, illustrating the trade-off between sensitivity and specificity. ( **A** ) *Mycobacterium tuberculosis* ; ( **B** ) cytomegalovirus (CMV); ( **C** ) arboviruses; ( **D** ) other viruses; ( **E** ) *Neisseria meningitidis* ; ( **F** ) *Treponema pallidum* ; ( **G** ) other bacteria; ( **H** ) varicella-zoster virus (VZV); ( **I** ) coinfections; ( **J** ) *Cryptococcus* spp.; ( **K** ) *Streptococcus pneumoniae* .

### Correlation between ADA levels and other laboratory data

A strong positive correlation was observed between CSF-ADA levels and CSF protein concentration (rs = 0.3009, *p* < 0.0003). In contrast, a weak negative correlation was found between CSF-ADA and CSF-glucose levels (rs = −0.1895, *p* < 0.0254). No other significant correlations were identified between CSF-ADA levels and serum parameters.

### Comparison of CSF-ADA levels in TBM and other infectious etiologies

Among infectious etiologies (mycobacterial, bacterial, viral and fungal groups), TBM presented the highest CSF-ADA levels. In the TBM group ( *n* = 45), the mean CSF-ADA concentration was 9.62 ± 8.51 (median, 8.0 [interquartile range, 4.7-11.3]; range, 0.6-46.8) U/L. The difference between the *M. tuberculosis* group ( *n* = 45) and the other infectious etiologies group ( *n* = 121) was assessed using the Mann-Whitney *U* test and was statistically significant ( *p* = 0.0007; Figure 5 ). When the infectious etiologies were analyzed separately, the Kruskal-Wallis test demonstrated a significant overall difference among the *M. tuberculosis* ( *n* = 45), bacterial ( *n* = 52), viral ( *n* = 25), fungal ( *n* = 22), and coinfection ( *n* = 22) groups ( *p* = 0.0065). Post hoc pairwise comparisons showed that CSF-ADA levels were significantly higher in the *M. tuberculosis* group than in the bacterial (median, 8.0 vs 3.6 U/L) and viral groups (median, 8.0 vs 2.9 U/L) ( Table 3 ).

**Figure 5:**
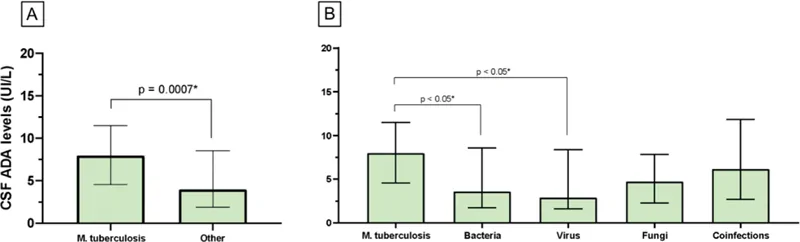
Comparison of CSF-ADA levels across infectious etiologies after exclusion of the non-infectious control group. ( **A** ) Comparison of tuberculous meningitis (TBM) with all other infectious etiologies combined. ( **B** ) Pairwise comparisons between TBM and each individual infectious etiology group.

**Table 3:** Summary statistics of CSF-ADA levels according to CNS infection subgroups

Group Total ( *n* = 166)	Mycobacteria ( *n* = 45)	Other infection ^a^ ( *n* = 121)	Bacteria ( *n* = 52)	Virus ( *n* = 25)	Fungi ( *n* = 22)	Coinfection ( *n* = 22)
Minimum	0.6	0.2	0.2	0.3	0.4	0.3
Maximum	46.8	58.5	58.5	38.1	15.8	27.7
Median	8.0	4.0	3.6	2.9	4.75	6.2
First quartile (Q1)	4.7	2	1.775	1.6	2.375	2.925
Third quartile (Q3)	11.3	8.5	8.525	7.6	7.325	10.4
Mean	9.6222	6.5521	6.4865	6.092	5.7727	8.0091
Standard deviation	8.5094	7.6933	8.8493	8.0097	4.5236	7.15

Abbreviations: CNS, central nervous system; CSF-ADA, cerebrospinal fluid adenosine deaminase.

Notes: *Kruskal-Wallis test: *p* -value = 0.0065, H = 14.6, degrees of freedom = 4.

1 Other infectious etiologies comprise the bacterial, viral, fungal and coinfection groups.

### Comparison stratified by the HIV status

A significant difference was observed between the VZV subgroup and the HIV versus non-HIV groups ( *p* = 0.0428, U = 2.5). The median CSF-ADA levels were 5.8 and 1.7 U/L in the HIV and non-HIV groups, respectively. Table 4 presents a comparison of all infectious etiologies stratified by HIV status.

**Table 4:** CSF-ADA levels among infectious etiologies according to HIV status

		n	Min	Max	Md	Q1	Q3	Mean	SD	*p* -value
***Mycobacterium tuberculosis***	HIV	35	0.6	46.8	6.3	4.4	10.1	9.0	8.5	0.1043
	Not	10	1.6	32.7	10.3	8.3	12.4	12.0	8.4	
***Streptococcus pneumoniae***	HIV	6	1.7	17.0	6.8	4.7	8.4	7.5	5.3	0.1415
	Not	17	0.6	15.3	2.7	1.8	5.8	4.5	4.1	
***Neisseria meningitidis***	HIV	4	0.7	58.5	11.7	4.5	27.8	20.6	26.2	0.5708
	Not	7	1.7	15.4	8.5	2.1	12.1	7.7	5.7	
***Treponema pallidum***	HIV	11	0.2	12.2	2.2	1.1	3.6	3.6	3.9	0.8358
	Not	2	1.5	7.0	4.3	2.9	5.6	4.3	3.9	
**Other bacteria** a	HIV	0								n/a
	Not	5	0.6	17.4	5.5	2.2	5.9	6.3	6.6	
**Cytomegalovirus**	HIV	6	0.3	38.1	7.8	1.7	12.7	11.3	14.2	n/a
	Not	1	4.0	4.0	4.0	4.0	4.0	4.0		
**Varicella-zoster virus**	HIV	4	1.7	16.5	5.8	3.4	9.8	7.4	6.5	0.0428*
	Not	6	1.4	2.9	1.7	1.6	2.5	2.0	0.6	
**Arboviruses**	HIV	0								n/a
	Not	4	0.3	9.1	6.7	4.7	7.7	5.7	3.8	
**Other viruses** b	HIV	2	1.8	11.2	6.5	4.2	8.9	6.5	6.6	n/a
	Not	2	1.0	2.0	1.5	1.3	1.8	1.5	0.7	
***Cryptococcus*** **sp.**	HIV	17	0.7	15.8	3.8	2.3	5.9	5.3	4.1	0.5208
	Not	3	0.4	6.2	4.3	2.4	5.3	3.6	3.0	
***Histoplasma capsulatum***	HIV	2	10.7	15.6	13.2	11.9	14.4	13.2	3.5	n/a
	Not	0								
**Coinfections**	HIV	17	2.3	27.7	7.6	4.5	14.3	9.9	7.1	0.5208
	Not	5	0.3	4.0	1.1	0.8	2.0	1.6	1.5	

Abbreviations: CSF-ADA, cerebrospinal fluid adenosine deaminase; HIV, human immunodeficiency virus; Max, maximum; Md, median; Min, minimum; n/a, not available; Q1, first quartile; Q3, third quartile; ROC, receiver operating characteristic; SD, standard deviation.

Notes: Mann-Whitney U test. * *p* -value < 0.05.

a *Streptococcus agalactiae* , *Streptococcus equi* , *Streptococcus suis* , *Streptococcus salivarius* and *Leptospira interrogans* .

b Herpes simplex virus type 1, herpes simplex virus type 2, human herpesvirus type 6 and enterovirus.

## DISCUSSION

This retrospective single-center diagnostic accuracy study evaluated the utility of CSF-ADA in microbiologically confirmed CNS infections. Compared with the control group ( *n* = 40), two subgroups demonstrated significantly elevated CSF-ADA levels: *M. tuberculosis* and *N. meningitidis* . The ADA is a metabolic enzyme that is predominantly active in lymphoid tissues and mononuclear cells. 12 It catalyzes the irreversible deamination of adenosine and 2'-deoxyadenosine into inosine and 2'-deoxyinosine 12 ; moreover, it participates in T-cell maturation and Th1 immune responses, which may explain its elevation in inflammatory CNS diseases with T-cell-mediated immunity. 13 14 Although the precise underlying mechanisms remain unclear, some studies suggest that ADA participates in a costimulatory interaction with CD26 to promote a Th1 response, which may theoretically explain its elevation in such conditions. 14 This study revealed a positive correlation between CSF-ADA levels and elevated CSF protein, which is a well-known marker of CNS inflammation, as well as a negative correlation between high CSF-ADA levels and low CSF glucose, possibly owing to consumption by bacterial or mycobacterial infections. The absence of correlation with lymphocyte and neutrophil counts ( *p* > 0.05) in our study does not prove that ADA levels are dependent on the predominant cell type but rather on the overall intensity of the inflammatory response.

In this study, we established ADA cutoff values for the *M. tuberculosis* (5.5 IU/L) and coinfection (7.3 IU/L) subgroups. For *M. tuberculosis* , the CSF-ADA test showed a sensitivity of 61.2% and specificity of 72.4%, whereas in the coinfection subgroup, CSF-ADA sensitivity and specificity were 50% and 82.8%, respectively. Several systematic reviews and meta-analyses have attempted to establish an optimal cutoff value and evaluate the clinical utility of CSF-ADA levels for the diagnosis of TBM. 2 4 15 16 The largest and most recent of these, which included 43 studies involving 1653 patients with TBM and 3417 controls, reported a sensitivity of 0.86, specificity of 0.89, positive likelihood ratio of 7.70, and negative likelihood ratio of 0.15, supporting good diagnostic accuracy with a cutoff value of 10 U/L ^2^ . In our study, the ADA cutoff value for TBM was 5.5 IU/L, which is lower than that reported in previous studies, including those involving HIV-infected individuals. In a study of 417 CSF samples from HIV-positive patients were analyzed, an optimal ADA cutoff of 8.5 IU/L was identified for the diagnosis of TBM, and yielded a sensitivity of 57%, specificity of 87% and an area under the curve (AUC) of 0.747. 17 Another study from India reported a similar CSF-ADA cutoff of 6.0 IU/L in HIV-infected patients, with a fair sensitivity of 75% and high specificity of 95.8%. 18 Interestingly, 33% of patients with TBM exhibited CSF-ADA levels below the established cutoff value of 5.5 IU/L. We hypothesize that the widespread availability of Xpert Ultra and the implementation of early lumbar puncture may facilitate earlier diagnosis, thereby identifying patients in the initial stages of infection—when the inflammatory response is less pronounced—and ultimately lead to lower CSF-ADA levels.

A significant proportion of patients in our study were co-infected with HIV. 19 Notably, HIV infection, HIV-associated neurological disorders and progressive multifocal leukoencephalopathy are usually not associated with elevated CSF-ADA levels. Reports of elevated CSF-ADA levels in HIV-infected individuals in the absence of opportunistic CNS infections are rare. 19 20 In addition, many CNS infections have immunosuppression as a key predisposing factor. The HIV infection impairs cell-mediated immunity by depleting CD4+ T-cells and increasing susceptibility to opportunistic CNS infections, such as CMV encephalitis, cryptococcal meningitis and toxoplasmosis. 21

Profound immunosuppression caused by HIV facilitates the reactivation of latent infections and impairs the clearance of newly acquired pathogens from the CNS. 21 Although no data are currently available on the effect of HIV infection on CSF-ADA levels in CNS diseases, the HIV status does not appear to compromise the diagnostic performance of ADA in pleural tuberculosis. 22 23 In our cohort, VZV infection was the only CNS infection associated with a significant difference in CSF-ADA levels according to the HIV status—a finding that, to our knowledge, has not been reported previously and warrants further investigation. No statistically significant differences were observed for the remaining CNS infections. Current international guidelines for TBM do not recommend the routine use of the CSF-ADA level as a diagnostic tool. 24 However, these guidelines have not been updated since 2009 and do not reflect the findings from more recent studies exploring the expanded diagnostic potential of ADA in this setting. 2 The reduced specificity likely reflects the wide range of CNS infections in HIV-positive patients rather than a flaw in ADA measurement. Interestingly, our data indicate that CSF-ADA may help diagnose both TBM and meningococcal meningitis, especially in settings with a high prevalence of HIV, and may therefore provide valuable clinical insights for regions with endemicity, such as Brazil.

Only a few cases with elevated CSF-ADA levels in cryptococcosis have been published. 25 26 27 28 Although CSF-ADA elevation is rarely documented in cryptococcal CNS infection, it is more frequently observed in pleural disease. 29 30 In our study, ADA levels in cryptococcal infection were low (cutoff, 4.5 IU/L) and not statistically significant. In contrast, other fungal infections, such as CNS histoplasmosis, showed markedly elevated ADA levels ( *n* = 2; median, 13.15 IU/L), despite being underrepresented. Given the scarcity of data on the CSF-ADA level in fungal CNS infections such as histoplasmosis, further research is needed to clarify the underlying mechanisms and potential diagnostic utility.

A notable finding of this study was the elevated CSF-ADA levels in the coinfection subgroup. This may be partially explained by the considerable presence of *M. tuberculosis* , which was identified in 8 of the 22 patients (36.4%), and is a well-established cause of elevated ADA levels. However, a diverse range of other pathogens, including VZV, CMV and *T. pallidum* , contributed to this subgroup. Interestingly, a study from Spain among patients with VZV-induced meningitis or encephalitis ( *n* = 6) reported unexpectedly high CSF-ADA levels, exceeding typical values seen in viral infections, among patients with VZV-related meningitis (mean, 13.9 ± 9.68 IU/L). 31 Nevertheless, in our analysis, this elevation pattern was not reproduced when VZV was assessed independently.

Another important finding was the significant increase in CSF-ADA levels in *N. meningitidis* cases ( *n* = 11; *p* = 0.0400) compared with the control group. Notably, the highest ADA value in our study (58.5 IU/L) was observed in this subgroup. Although several studies have compared CSF-ADA levels in TBM versus non-TBM meningitis—and frequently demonstrated higher ADA in bacterial than viral etiologies—, the specific role of ADA in individual bacterial pathogens, such as *N. meningitidis* , remains poorly characterized. To date, no study has directly evaluated CSF-ADA levels in patients with meningococcal meningitis. An investigation that assessed serum ADA levels in patients with meningococcal pneumonia reported significantly decreased levels (mean: 3.5 ± 3.4 IU/L, *p* < 0.001) compared with controls and cases with *S. pneumoniae* infection. 32 The elevated CSF-ADA levels that were observed in our patients with meningococcal meningitis may reflect robust immune activation, particularly the recruitment of neutrophils and monocytes into the subarachnoid space. 2 This intense cellular response could drive increased ADA production via enhanced purine metabolism during inflammation. 2

This study has some limitations. First, although most patients in the ADA control group had noninfectious conditions, such as psychiatric disorders, vascular diseases, or migraine, the complete exclusion of occult infections cannot be guaranteed. However, this risk is minimized by the routine use of molecular diagnostics and culture testing in the majority of cases, as well as the absence of inflammatory findings (such as elevated protein or pleocytosis) in CSF analyses. 33 Second, the arbovirus subgroup included multiple distinct arboviral infections, precluding pathogen-specific analysis. Third, the coinfection group was highly heterogeneous, with some patients presenting with up to three concurrent CNS infections. This overlap may have contributed to elevated ADA levels due to a heightened immune response; however, the heterogeneity precludes attribution to any specific pathogen. Fourth, despite the overall robust sample size, the inclusion of multiple etiologies resulted in small subgroup sizes, reducing the statistical power for certain analyses. This was particularly evident in the *H. capsulatum* group. Nevertheless, *H. capsulatum* is a rare cause of meningitis, and data on CSF-ADA levels in this population remain scarce. Furthermore, one patient with *N. meningitidis* infection exhibited disproportionately high CSF-ADA levels, which may have influenced the statistical analysis. Even so, the diagnosis was confirmed using a multiplex polymerase chain reaction (PCR) panel. Larger prospective studies are needed to validate these findings and address potential biases.

In conclusion, CSF-ADA may be a valuable tool to aid in the differentiation of CNS infections when interpreted alongside other biochemical laboratory markers, even in populations with a high HIV-infection prevalence. However, clinicians, neurologists and infectious disease specialists should be aware that elevated ADA levels are not exclusive to TBM but may also be observed in other infectious etiologies, such as meningococcal disease, fungal infections and coinfections. In certain cases, meningococcemia may lead to elevated CSF-ADA levels, and potentially complicate the differential diagnosis with TBM. In summary, our study provides important data on a wide range of CNS infectious etiologies, including tropical diseases such as arboviruses, and their correlation with CSF-ADA levels. Nonetheless, caution is warranted when interpreting ADA levels in the CNS, and further studies are necessary to better define the diagnostic value of CSF-ADA in CNS infections.
